# Zinc Metalloproteinases and Amyloid Beta-Peptide Metabolism: The Positive Side of Proteolysis in Alzheimer's Disease

**DOI:** 10.1155/2011/721463

**Published:** 2010-09-30

**Authors:** Mallory Gough, Catherine Parr-Sturgess, Edward Parkin

**Affiliations:** Division of Biomedical and Life Sciences, School of Health and Medicine, Lancaster University, Lancaster LA1 4YQ, UK

## Abstract

Alzheimer's disease is a neurodegenerative condition characterized by an accumulation of toxic amyloid beta- (A*β*-)peptides in the brain causing progressive neuronal death. A*β*-peptides are produced by aspartyl proteinase-mediated cleavage of the larger amyloid precursor protein (APP). In contrast to this detrimental “amyloidogenic” form of proteolysis, a range of zinc metalloproteinases can process APP via an alternative “nonamyloidogenic” pathway in which the protein is cleaved within its A*β* region thereby precluding the formation of intact A*β*-peptides. In addition, other members of the zinc metalloproteinase family can degrade preformed A*β*-peptides. As such, the zinc metalloproteinases, collectively, are key to downregulating A*β* generation and enhancing its degradation. It is the role of zinc metalloproteinases in this “positive side of proteolysis in Alzheimer's disease” that is discussed in the current paper.

## 1. Introduction

Alzheimer's disease (AD) is the leading form of dementia in the elderly, accounting for some two-thirds of all cases and exhibiting a prevalence of 5% in individuals older than 65 years. The disease is clinically characterized by a progressive cognitive impairment, including impaired decision making, orientation and judgement often accompanied, in the later stages, by psychobehavioural disturbances and language impairment. AD was originally described in 1906 by the German psychiatrist and neuropathologist, Alois Alzheimer, but it was his coworker, Emil Kraepelin who first coined the term “Alzheimer's disease” [1]. What Alzheimer described were what we now know as the two major pathological hallmarks in the brains of AD-afflicted individuals, amyloid (also known as senile) plaques and neurofibrillary tangles (NFTs) [2]. At the molecular level, NFTs are composed of tau, a microtubule-associated protein which, in AD, becomes hyperphosphorylated and forms insoluble intracellular fibrils [3]. Amyloid plaques, on the other hand, are extracellular structures composed of 38–43 amino acid peptides called amyloid beta (A*β*)-peptides.

Although it is important to appreciate that AD is a multifactorial disease [4], a key theory as to disease causation is that of the “amyloid cascade hypothesis” whereby A*β*-peptides are the leading cause of toxicity to neurons [5]. The initial version of the hypothesis proposed that mature amyloid fibrils and plaques in the brain were responsible for the observed neurotoxicity, but more recent incarnations point towards the earlier stage, smaller soluble A*β* aggregates being the primary cause of AD [6, 7]. Whatever the case, it is clear that an increase in A*β* in the brain has a role to play in AD pathogenesis. 

Proteolysis dictates both the level of A*β*-peptides generated in the first instance and the rate at which they are degraded in the second. As such, a fine balancing act of proteolytic enzyme activities is at play in the brain keeping A*β* levels in check. Although a range of proteinase classes are involved directly or indirectly in the metabolism of A*β*, most notably it is the zinc metalloproteinases that are key to downregulating A*β* generation and enhancing its degradation. It is the role of zinc metalloproteinases in this “positive side of proteolysis in Alzheimer's disease” that will be discussed in the current paper.

## 2. A*β*-Peptide Generation: A Balancing Act between Nonamyloidogenic and Amyloidogenic Proteolysis

**Figure 1:**
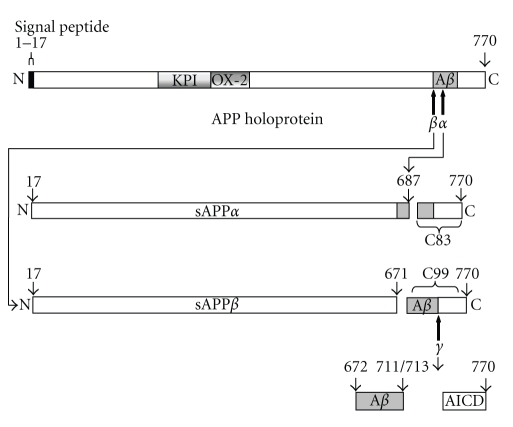
Proteolysis of the amyloid precursor protein (APP). APP can be cleaved by two alternative proteolytic pathways, the amyloidogenic and nonamyloidogenic pathways. It is the balance between these two pathways which dictates the levels of A*β*-peptides generated. KPI: Kunitz-type serine proteinase inhibitor domain; OX-2: OX-2 domain; A*β*: amyloid beta; AICD: APP intracellular domain.

A*β*-peptides are generated from a much larger precursor protein, the amyloid precursor protein (APP), a ubiquitous type I cell surface protein of as yet unknown physiological function (Figure 1) [8]. The protein exists in multiple isoforms as a result of alternative splicing of the 19 exons encoded by the *APP* gene [9]. Exon 7 encodes for a 57-amino acid region with considerable homology to a Kunitz-type serine protease inhibitor (KPI) and is present in the larger APP_770_ and APP_751_ isoforms, but absent from the smaller APP_695_ protein. 

The APP holoprotein can be proteolytically degraded via an amyloidogenic pathway (Figure 1 and reviewed in [10]) which involves initial cleavage by *β*-secretase (*β*-site APP-cleaving enzyme 1; BACE1) to generate a soluble N-terminal fragment termed sAPP*β* along with a C-terminal membrane-associated fragment (CTF) of 99 amino acids. The C99 fragment is then further processed by a *γ*-secretase complex producing the A*β*-peptides and the APP intracellular domain (AICD) [11]. Alternatively, APP can be processed via a nonamyloidogenic route involving *α*-secretase cleavage within the A*β* domain (reviewed in [12]). This latter cleavage occurs on the C-terminal side of Lys687 (APP_770_ numbering) [13] and precludes A*β*-peptide formation, generating instead a soluble APP ectodomain (sAPP*α*) along with a CTF of 83 amino acids (C83) (Figure 1).

## 3. Zinc Metalloproteinases in the Prevention of A*β* Generation

The *α*-secretase-mediated nonamyloidogenic processing of APP (Figure 1) represents a positive side of proteolysis in AD in that it precludes formation of intact A*β*-peptides, that is, there is a reciprocal relationship between nonamyloidogenic and amyloidogenic APP processing. Skovronsky et al. [14] demonstrated that the activation of *α*-secretase APP processing using phorbol esters in CHO cells stably transfected with APP_695_ led to a reduction in both sAPP*β* and A*β* production. At the *in vivo* level, the overexpression of *α*-secretase activity in APP ([V717I]) transgenic mice increased sAPP*α* generation with a concomitant reduction in the formation of A*β*-peptides [15]. Proteolysis of APP by *α*-secretases may also be considered a positive event in that the sAPP*α* generated enhances the proliferation of both nonneuronal and neuronal precursor cells [16–18], stimulates neurite extension in immortalized neuronal cell lines [19], modulates transmission at the synapse, and is neuroprotective against ischemic, excitotoxic, and traumatic brain injuries [20–24]. *In vivo*, the intracerebral injection of sAPP*α* has been shown to enhance memory performance in adult rats [25], and a truncated APP deletion variant corresponding to sAPP*α* has been shown to rescue anatomical, behavioural, and electrophysiological abnormalities in APP-deficient mice [26] further underlining the physiological importance of sAPP*α* generation. 

**Figure 2:**
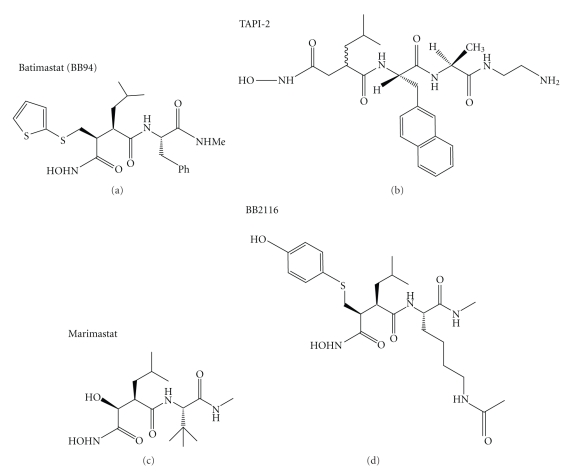
Structures of hydroxamic acid-based zinc metalloproteinase inhibitors.

Given the positive aspects of nonamyloidogenic APP processing, the identity of the *α*-secretase was of great interest to Alzheimer's researchers. The first clue in this respect came when it was demonstrated that the *α*-secretase was an integral membrane proteinase with its preference for the Lys687-Leu688 peptide bond of APP being dictated more by the proximity of the bond to the membrane than the absolute amino acid specificity of the cleavage site [27, 28]. Using a range of class-specific proteinase inhibitors, it was subsequently demonstrated that only the zinc chelator, 1,10-phenanthroline, caused significant inhibition of APP release from crude membrane preparations in a cell-free system [29] suggesting for the first time that one or more zinc metalloproteinases constituted the *α*-secretase activity. A number of groups later reported that the active site-directed zinc chelating compounds batimastat and TAPI-2 (Figure 2) inhibited the release of sAPP*α* into the conditioned media of a variety of cell lines [29–31]. 

A range of studies demonstrated that the zinc metalloproteinase activity responsible for generating sAPP*α* was similar to that responsible for proteolytically “shedding” a number of other substrate proteins from the cell surface. For example, Parvathy et al. [30] compared the shedding of APP to that of the angiotensin-converting enzyme (ACE; EC 3.4.15.1) demonstrating that the release of both proteins from transfected IMR-32 cells was inhibited by the hydroxamic acid-based compounds batimastat, marimastat, and BB2116 (Figure 2) with IC_50_ values in the low micromolar range. In addition, Parkin et al. [32] demonstrated that a range of hydroxamic acid-based compounds failed to discriminate between the proteinases responsible for shedding APP and the cellular form of the human prion protein, and it has subsequently been demonstrated that both proteins are indeed shed by the same enzyme [33]. Indeed it has become apparent that the *α*-secretase activity is constituted by one or more members of the ADAM (a disintegrin and metalloproteinase) family of zinc metalloproteinases now known to be responsible for shedding a multitude of cell surface integral membrane proteins (reviewed in [34]).

### 3.1. The ADAMs Family of Zinc Metalloproteinases as APP *α*-Secretases

**Figure 3:**
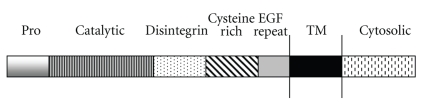
The domain structure of a disintegrin and metalloproteinases (ADAMs).

The a disintegrin and metalloproteinases (ADAMs) are zinc metalloproteinases belonging to the metzincin super family. They are type I transmembrane glycoproteins that constitute a family of some forty members [34] which have a common modular ectodomain structure consisting of an N-terminal prodomain, a catalytic domain, a disintegrin domain, and a cysteine-rich domain (Figure 3). The ADAM prodomain appears to be essential for protein maturation maintaining the metalloproteinase site of the newly synthesized protein in an inactive conformation by way of a cysteine switch mechanism [35, 36] until the domain is cleaved by prohormone convertases (PCs) such as furin and PC7 [15, 35, 37, 38]. Furthermore this domain seems to be essential for the correct folding of at least two ADAM family members (ADAM10 and 17) as its deletion resulted in catalytically inactive forms of the proteins [35, 39]; in the case of ADAM10, catalytic activity of the enzyme could be rescued by cotransfection of the inactive construct with one encoding the prodomain alone [35].

The prodomain is followed by a region which, in seventeen of the known ADAMs, contains the consensus sequence, HEXGHXXGXXHD, predicted to be the catalytic site of an active zinc metalloproteinase [34]. This catalytic domain is followed by the disintegrin domain, a 60–90-amino acid residue stretch bearing limited sequence homology to the disintegrin proteins isolated from snake venom and which participates in various cell-cell adhesion events [40–45]. Finally, the cytosolic domains of the ADAMs are involved in the binding of a wide range of adaptor proteins which may influence maturation, trafficking, or proteolytic activity of the enzymes [46].

It is becoming increasingly apparent that the *α*-secretase activity is constituted by more than one ADAM family member and that the profile of these zinc-dependent APP-cleaving enzymes differs considerably between different cell lines.

#### 3.1.1. ADAM10

ADAM10 (EC 3.4.24.81) was first identified in 1989 as a peptide sequence purified from bovine brain myelin membrane preparations and referred to as MADM (Mammalian Disintegrin Metalloprotease) [47]. Following cloning, the first catalytic activity assigned to the enzyme was its ability to degrade myelin basic protein (MBP) [48]. Like other ADAM family members, ADAM10 has a modular ectodomain structure and is synthesized as an inactive zymogen being activated only after enzymatic removal of its prodomain [35]. In fact the recombinant ADAM10 prodomain purified from *E. coli* is a potent and selective inhibitor of the enzyme *in vitro * [49]. The catalytic domain of ADAM10 contains the zinc-binding consensus motif, HEXGHXXGXXHD, whilst glycosylation sites containing high-mannose and complex *N*-glycans are located both in the catalytic and disintegrin domains [50]. Furthermore, an N439 mutation at the *N*-glycosylation site of the enzyme has been shown to increase the susceptibility of ADAM10 to proteolytic degradation [50]. 

The gene locus for ADAM10 in humans is on chromosome 15 (15q21.3–q23) and chromosome 9 in mice [51, 52], and both sequences are approximately 160 kb in length. The core promoter region in the human gene has been identified by deletion analysis as nucleotides −508 to −300 bp which represents a TATA-less promoter containing functional binding sites for USF, Sp1, and retinoic acid receptors [53, 54]. In fact, the NAD-dependant deacetylase SIRT1 directly activates transcription of the ADAM10 gene possibly via the deacetylation and coactivation of the retinoic acid receptor beta [55].

Given the fact that the original reported substrate for ADAM10 (MBP) is a cytosolic protein and ADAM10 itself is a type I membrane protein, it is unlikely that this substrate is one of physiological relevance. However, it is now known that the zinc metalloproteinase activity of ADAM10 is responsible for proteolytically shedding more than forty integral membrane substrates from the cell surface including growth factors (e.g., betacellulin and epidermal growth factor), adhesion proteins (e.g., L1), other proteinases (e.g., BACE1), and a range of other substrates including the human prion protein [33] along with the Notch receptor and one of its ligands, Delta [56].

Although it is now known that quite a range of ADAMs may constitute the *α*-secretase activity, it is undoubtedly ADAM10 that has sparked most research interest as an APP cleaving enzyme. Lammich et al. [57] first demonstrated that bovine ADAM10 overexpressed in HEK cells enhanced the basal cleavage of APP. The authors further demonstrated that APP cleaving activity stimulated by treatment of the cells with phorbol ester (regulated cleavage) was also enhanced by ADAM10 suggesting that the enzyme had a role in both basal and regulated APP proteolysis. In addition, a dominant negative form of ADAM10 with a point mutation in the active site (E384A) inhibited the endogenous *α*-secretase activity in HEK cells [57]. Furin-deficient LoVo cells which are devoid of regulated *α*-secretase activity have also been used to demonstrate that overexpression of ADAM10 enhanced the basal production of sAPP*α* [38].

The involvement of ADAM10 in the zinc metalloproteinase-mediated cleavage of APP is also supported by studies using synthetic peptide substrates. An 18-mer peptide spanning the *α*-secretase cleavage site was cleaved by purified bovine kidney ADAM10 at the physiologically relevant *α*-secretase cleavage site (Lys16-Leu17 of the A*β* region) [57]. Intriguingly, the insertion of a naturally occurring APP mutation associated with cerebral haemorrhages due to amyloid angiopathy (A21G) [58] into a similar synthetic peptide substrate resulted in cleavage by ADAM10 at a slower rate than the wild-type sequence peptide. Finally, Amour et al. [59] demonstrated that an 11-mer peptide spanning the *α*-secretase cleavage site was cleaved by a recombinant soluble form of the human ADAM10 catalytic domain.

The *in vitro* cleavage of synthetic peptide substrates by an enzyme can often bear limited parallels to the cleavage of full-length physiological protein substrates. Unfortunately, the study of ADAM10-mediated APP cleavage *in vivo* was, for some time, limited by the embryonic and perinatal lethality of ADAM10 knockout mice [60]. However, in 2004, human APP overexpressing mice were crossed with ADAM10 transgenic animals, and the resultant progeny demonstrated enhanced production of sAPP*α* in their brains with a concomitant reduction of A*β* [15]. In contrast, the crossing of a dominant-negative ADAM10 mutant mouse with an APP transgenic animal had the opposite effect, inhibiting *α*-secretase cleavage of APP with a slight increase in A*β* production. These studies clearly demonstrated that the zinc metalloproteinase activity of ADAM10 was capable of competing for APP *in vivo* with enzymes of the amyloidogenic pathway.

A number of groups have demonstrated a link between ADAM10 trafficking and maturation, and the zinc metalloproteinase activity responsible for the generation of sAPP*α*. The overexpression of PC7 and furin in HEK cells transfected with ADAM10 resulted in a greater increase in sAPP*α* production than when either enzyme was transfected into cells expressing only endogenous ADAM10 [35] suggesting that it was the removal specifically of the ADAM10 prodomain and subsequent activation of the enzyme that correlated with the level of *α*-secretase activity. The maturation of ADAM10 can also be chemically manipulated so as to increase sAPP*α* production. Epigallocatechin-3-gallate (EGCG), a constituent of green tea, increased levels of the mature 60 kDa form of ADAM10 via a mechanism of action specific to ADAM10 (the increase in sAPP*α*  production brought about by EGCG was only impaired in cells treated with ADAM10 siRNA and not those treated with siRNA targeted to ADAMs 9 and 17) [61]. The acetylcholinesterase inhibitor, donepezil, also concomitantly increased sAPP*α* production and the amount of mature ADAM10 in the membrane fraction of SH-SY5Y cells, an effect which could be prevented by pretreatment of the cells with tunicamycin or brefeldin suggesting a mechanism of action involving the trafficking/maturation of the ADAM10 protein [62]. Finally, it seems that other proteins required for the trafficking of ADAM10 can affect sAPP*α* production. Marcello et al. [63] demonstrated that synapse-associated protein-97 (SAP97), a protein involved in dynamic trafficking of proteins to the excitory synapse, was responsible for directing ADAM10 to the postsynaptic membrane via a direct interaction through its Src homology 3 domain. The authors more recently reported [64] that ADAM10 contains an arginine-rich ((723)RRR) sequence in its cytosolic domain responsible for retaining the protein in the endoplasmic reticulum (ER) and that, when the sequence was mutated, protein exit from the ER occurred. However, SAP97 was not thought to be involved in regulating this ER exit suggesting the role of alternative protein binding partners in this event.

#### 3.1.2. ADAM17

ADAM17 (EC 3.4.24.86) was originally identified as the proteinase responsible for shedding the inflammatory cytokine tumour necrosis factor (TNF)-*α* from its membrane-associated precursor TNF [65, 66] and is, therefore, alternatively referred to as tumour necrosis factor-*α* converting enzyme (TACE). ADAM17 is a type I integral membrane zinc metalloproteinase, the catalytic domain of which has been cocrystallised with the hydroxamic acid-based inhibitor, Immunex compound 3, and the structure solved at 2.0 *Ǻ*  resolution [67]. 

Like ADAM10, ADAM17 has been implicated in the proteolysis of a large range of cell surface proteins of diverse functions (reviewed in [68]). In relation to the cleavage of APP though, the basal shedding of the protein was unaffected in primary embryonic fibroblasts derived from ADAM17 knockout mice whereas the phorbol ester regulated cleavage was found to be deficient [69, 70]. Similarly, the ADAM17 inhibitor, CP-661631, inhibited regulated but not constitutive sAPP*α* secretion from human primary neuron cultures without affecting A*β*-peptide production [71]. In light of the latter observation, it is perhaps of little surprise that it has more recently been shown that ADAM10 and not ADAM17 or 9 is the physiologically relevant basal *α*-secretase activity in primary neurons [72]. However, there is considerable evidence to suggest that the enzyme is involved in basal APP shedding in various other cell lines. Only constitutive and not muscarine-regulated APP cleavage was enhanced in HEK cells transfected with ADAM17 [73]. Similarly, Hiraoka et al. [74] demonstrated that overexpression of ADAM17 in COS7 cells lead to a significant increase in basal sAPP*α* secretion. In contrast Asai et al. [75] used short interfering RNAs (siRNAs) to deplete ADAM17 protein levels in human glioblastoma A172 cells and subsequently demonstrated that the proteinase was involved in both the constitutive and regulated endogenous *α*-secretase processing of APP. Thus, data derived from *in vitro* cell culture studies seem to suggest that ADAM17 is generally involved in the regulated processing of APP but that its involvement in constitutive sAPP*α* production is somewhat more cell type-dependent.

Studies using synthetic peptide substrates offer less convincing proof that ADAM17 is involved in the *α*-secretase processing of APP. Buxbaum et al. [76] reported that recombinant ADAM17 cleaved a synthetic substrate spanning the *α*-secretase cleavage site in APP at the Lys16-Leu17 (A*β*-peptide numbering) bond. However, the authors did not present data pertaining to the kinetics of the cleavage. In contrast, Mohan et al. [77] examined the kinetics of cleavage of a synthetic TNF*α* substrate by recombinant and native ADAM17 and compared the cleavage to that of several other proposed ADAM17 substrates, including APP. The authors found that ADAM17 efficiently cleaved the TNF*α* substrate but only cleaved the APP-derived peptide substrate at high enzyme concentrations and extended reaction times suggesting that APP might be a poor substrate for the enzyme *in vivo*.

#### 3.1.3. ADAM9 and ADAM15

The cDNA encoding the zinc metalloproteinase ADAM9 (EC 3.4.24.B9) was isolated originally from a murine lung cDNA library [78]. Initially it was thought that the enzyme might be directly responsible, at least in part, for the *α*-secretase-mediated processing of APP. The overexpression of both ADAM9 and APP in COS cells led to an increase in the phorbol ester-regulated generation of sAPP*α* [79]. The authors in this study did not, however, determine the exact site of cleavage by ADAM9 leaving open the possibility that the enzyme had a bond specificity other than at the “regular” *α*-secretase cleavage site (Lys16-Leu17). This latter point is certainly a possibility given the fact that a soluble recombinant form of ADAM9 has been shown to cleave a 12-mer synthetic peptide spanning the APP *α*-secretase cleavage site at the His14-Gln15 bond within the A*β* region [80]. However, a secreted form of ADAM9, when coexpressed with APP in CHO cells, has been shown to enhance phorbol ester-regulated APP cleavage at Lys16-Leu17 [81].

Given the fact that promoter polymorphisms enhancing ADAM9 transcription are protective against sporadic AD [82], there does seem to be a connection between this zinc metalloproteinase and the disease. However, research into the possibility of a physiologically relevant direct cleavage of APP by ADAM9 has all but petered out in recent years. Instead, it now seems that ADAM9 influences APP processing in an indirect manner via an effect on ADAM10 as the transient overexpression of the former enzyme in ADAM10^−/−^ fibroblasts, in contrast to wild-type fibroblasts, had no effect on sAPP*α* production [83]. More recently it has been demonstrated in our laboratory that ADAM9 actually sheds ADAM10 from the cell surface [84] although it remains unclear as to how this event would enhance *α*-secretase shedding of APP as we also demonstrated that a truncated soluble ADAM10 construct analogous to the shed form of the enzyme did not enhance sAPP*α* production despite being catalytically active against a synthetic peptide substrate. Following this observation Tousseyn et al. [85] demonstrated that both ADAM9 and ADAM15 are capable of shedding ADAM10 and presented evidence suggesting that the C-terminal membrane-associated ADAM10 fragment generated following cleavage by ADAM9 could be further processed to liberate an intracellular domain involved in nuclear signaling. This latter observation opens the possibility that ADAM9-mediated ADAM10 nuclear signaling may somehow enhance *α*-secretase cleavage of APP.

#### 3.1.4. Other ADAMs as APP *α*-Secretases

Although it now seems that ADAM10 is the physiologically relevant *α*-secretase in primary neurons [72], a number of additional ADAMs over and above ADAMs 9 and 17 have been implicated in APP processing. Zou et al. [86] demonstrated that ADAM33 (EC 3.4.24.-) could cleave a synthetic *α*-secretase substrate but that cleavage occurred at His14-Gln15 rather than Lys16-Leu17 and with quite low efficiency making it unlikely that the enzyme is a physiologically relevant *α*-secretase. In addition, Naus et al. [87] demonstrated that ADAM8 (EC 3.4.24.-) could cleave APP in HEK cells with a similar efficiency to that of ADAM10. Finally, Tanabe et al. [88] demonstrated that overexpression of ADAM19 (EC 3.4.24.-) in A172 cells enhanced constitutive sAPP*α* production and that siRNA depletion of the endogenous enzyme decreased sAPP*α* production by approximately 21%. However, it would seem that ADAM8 and ADAM19 are, at best, responsible for a very minor fraction of sAPP*α* generated in primary neurons given the major contribution of ADAM10 in this respect [72].

### 3.2. Nardilysin: An ADAM Activating Zinc Metalloendopeptidase

Nardilysin (N-arginine dibasic convertase; NRDc; EC 3.4.24.61) is a member of the inverzincin/M16 family of zinc metalloendopeptidases [89]. This family of enzymes have in common an approximately 200-amino acid conserved region containing the HXXEH binding motif of catalytic Zn^2+^. NRDc has been shown to enhance the constitutive shedding of sAPP*α* from COS7 cells transiently transfected with APP_WT_, an effect that was totally abolished by the hydroxamic acid-based metalloproteinase inhibitor TAPI-2 [74]. It now appears that NRDc enhances ADAM9, -10, and -17 activities regardless of the substrate being cleaved [90] via a mechanism of action which remains unclear but which does seem to involve a direct interaction of the proteins.

## 4. Zinc Metalloproteinases in the Degradation of A*β*

Just as ADAMs can prevent A*β* generation in the first instance, the proteolytic degradation of preformed A*β*-peptides represents another way by which to decrease the steady-state levels of these potentially neurotoxic peptides in the brain. Although proteinases from a number of different classes can degrade A*β*, by far the dominant class in this respect is that of the zinc metalloproteinases. 

### 4.1. Insulin Degrading Enzyme

The insulin degrading enzyme (IDE) (EC 3.4.24.56) (insulysin, insulin protease) is a 100–120 kDa ubiquitously expressed zinc metallopeptidase belonging to the M16A class of metalloendopeptidases characterised by an inverted HXXEH motif in the active site (as opposed to the HEXXH motif of other zinc metalloproteinases). The enzyme is evolutionarily conserved and possesses alternatively spliced and initiated variants [91]. IDE cleaves a broad range of physiologically active peptides including insulin, glucagon, and atrial natriuretic factor with its main physiological function being to regulate steady-state levels of peripheral insulin [92]. The structure of IDE resembles a clam shape consisting of four homologous domains, and the enzyme is dependent on an ATP-powered structural regulatory switch in order to permit substrate access to the active site [91].

As far as cleaving A*β* is concerned, IDE degrades A*β* in conditioned medium from neuronal cultures [93], and both rat and mouse IDE knockout animals exhibit enhanced brain A*β* levels [94, 95]. Conversely, the overexpression of IDE in transgenic mice results in drastically reduced brain A*β* levels and decreased plaque formation along with reduced AD-like cytopathology [96]. In addition to cleaving A*β*, it has been suggested that IDE might also cleave the APP intracellular domain as evidenced by increased AICD levels in the brains of IDE knockout mice [97]. However, the treatment of cell cultures with IDE inhibitors did not enhance AICD levels [98] suggesting that the direct cleavage of the fragment by IDE alone might not account for the enhanced AICD levels observed in IDE knockout animals. 

In AD-afflicted human brain, IDE is associated with senile plaques and A*β* deposits in microvessels [99, 100], and hippocampal levels of the enzyme are reduced in patients considered to be at high risk of developing AD [101]. At the genetic level, the risk of developing AD has been linked to variation within a 276 Kb region of chromosome 10 encoding, *interalia*, IDE [102]. Furthermore, decreased IDE catalytic activity but not expression was detected in the lymphoblasts of chromosome 10-linked AD family members [103].

### 4.2. Neutral Endopeptidase

Neutral endopeptidase (NEP) (EC. 3.4.24.11) (neprilysin, enkephalinase, endopeptidase 24.11, kidney brush border neutral proteinase, common acute lymphoblastic leukaemia antigen, and CD10) is an 85–93 kDa type II membrane protein belonging to the M13 class of zinc metalloendopeptidases [104]. The protein consists of a short N-terminal cytosolic region, a single transmembrane helix, and a large C-terminal ectodomain containing, *interalia*, the catalytic site with a conserved HEXXH motif participating in zinc coordination [91]. NEP exhibits an extensive substrate specificity cleaving many biologically active peptides including substance P, neuropeptide Y, enkephalin, and cholecystokinin; the enzyme, therefore, has extensive roles in neuropeptide signaling and the regulation of vascular tone [104].

The fact that NEP might be involved in A*β* degradation originally came to light when it was established that the enzyme could cleave A*β*,  but not full-length APP, *in vitro* [105]. However, it took a further five years to establish that NEP was involved in the *in vivo* degradation of A*β* when Iwata et al. [106] injected radioactive A*β*-peptides into rat brain and observed that their subsequent degradation was inhibited by compounds such as phosphoramidon and thiorphan. It was subsequently shown that NEP knockout mice exhibited significantly elevated levels of A*β* in their brains [107] and that, when APP transgenic mice were crossed with NEP-deficient animals, the resultant progeny exhibited impaired hippocampal synaptic plasticity and increased cognitive decline compared to APP transgenics with normal levels of NEP [108].

In human postmortem brain samples an increased amyloid plaque density is associated with decreased NEP immunoreactivity [109], and, in the brains of AD patients, NEP mRNA levels in the hippocampus and temporal gyrus were lower than those in the same regions of control brains [110]. Furthermore, an age-dependant decline in NEP levels has been demonstrated in both normal and AD brains [111].

At the genetic level, at least two single nucleotide polymorphisms (SNPs) located within introns of the NEP gene have been positively associated with AD [112], and an NEP GT-repeat polymorphism is also associated with the late-onset form of the disease [113]. Finally, reduced NEP expression in AD is associated with an APOE *ε*4 genotype which itself is a major risk factor for sporadic AD [114].

### 4.3. Endothelin Converting Enzymes 1 and 2

Endothelin converting enzymes (ECEs) (EC. 3.4.24.71) are members of the M13 group of proteins (type II integral membrane proteins with zinc metalloproteinase activity) and display several sequence and domain structure similarities to another member of this family, NEP (Section 4.2). ECEs cleave biologically inactive big endothelins to generate mature endothelins which act as vasoconstrictors [115]. 

Three isoforms of ECEs have been reported, ECE-1, ECE-2, and ECE-3, but only the former two have been implicated in A*β* degradation. Four alternative splice variants of ECE-1, localized both at the cell membrane and intracellularly, have been reported in humans (ECE-1a, ECE-1b, ECE-1c, and ECE-1d) [116, 117]. In contrast, ECE-2 is localized almost exclusively to the *trans*-Golgi network [115]. Eckman et al. [118] initially demonstrated that the metalloproteinase inhibitor phosphoramidon enhanced extracellular levels of A*β* in cultured cells through an inhibition of peptide degradation. The authors also showed that the overexpression of ECE-1 in CHO cells resulted in a 90% reduction of A*β* levels and that recombinant ECE-1 was capable of cleaving A*β* at multiple sites. The same group subsequently reported a significant increase in A*β* levels in the brains of ECE-1 and ECE-2 knockout mice [119] and also demonstrated even greater A*β* accumulation in the brains of combined ECE-1 and NEP knockout mice suggesting that both enzymes act in unison to degrade A*β* [120]. Interestingly, both ECE-1 and ECE-2 mRNA levels were enhanced when cell cultures were treated with A*β*  suggesting the existence of a feedback loop mechanism whereby increases in A*β* levels enhance its own degradation [121, 122]. 

At the genetic level, Funalot et al. [123] identified a C338A polymorphism in the regulatory region of the ECE-1b gene that was associated with increased transcriptional activity. The authors demonstrated that individuals homozygous for the A allele were at a reduced risk of developing AD. An additional study also revealed a protective role for the A allele within a cohort of Chinese subjects [121]. Furthermore, using microarray technology, Weeraratna et al. [124] observed that the most significantly down regulated gene in AD was that of ECE-2. The authors also presented immunohistochemical evidence demonstrating a reduction in ECE-2 protein expression in inferior parietal lobe tissue from AD brain.

### 4.4. Angiotensin Converting Enzyme

The angiotensin converting enzyme (ACE) (EC 3.4.15.1) is a type I integral membrane zinc-dependent dipeptidase that cleaves two vasoactive peptides, angiotensin I and bradykinin [125, 126]. Consequently, the enzyme plays an important role in the regulation of hypertension and in the development of vascular pathology and endothelium remodelling in some disease states [127, 128]. There are two distinct isoforms of mammalian ACE, somatic (sACE) and testis (tACE), both of which are transcribed from a single gene by the use of two alternative promoters [129]. Somatic ACE (180 kDa) consists of two identical catalytic domains (C- and N-domains), both bearing a functional zinc-dependent active site, whereas testis ACE consists of only a single domain corresponding to the C-terminal domain of sACE [128].

Of all the zinc metalloproteinases involved in A*β* degradation, there is perhaps most controversy surrounding ACE. The enzyme was originally shown to degrade A*β* *in vitro *cleaving the Asp7-Ser8 and Arg5-His6 peptide bonds [130], and, more recently, the A*β* degrading activity of ACE has been reported to be located specifically within the protein's N-domain [131]. One study reported a significant elevation of A*β* levels in the brains of mice treated with the ACE inhibitor, captopril [132]. Furthermore, the ACE inhibitor, perindopril, has recently been shown to improve cognitive performance in APP/presenilin-1 transgenic mice injected with A*β* [133]. However, for every study reporting a role of ACE in the prevention of AD, there is at least one other that presents negative date in this regard. For example, there are several studies which, using genetic or chemical inactivation of ACE *in vivo*, suggest that the enzyme does not have a substantial physiological role to play in the degradation of A*β* [106, 120, 134]. In this respect it is notable that another of the physiological substrates of ACE, angiotensin I, is converted to the potent vasoconstrictor ACE II. Consequently, both substrate and product are ligands for subtypes of androgen receptors, the regulation of which has also been implicated in AD [135]. The question arises, therefore, as to whether the effect of ACE inhibitors on A*β* levels is due to the inhibition of the enzyme's A*β* degrading activity or to alterations in the relative levels of angiotensins I and II and their abilities to interact with their cognate receptors.

### 4.5. Matrix Metalloproteinases

Matrix metalloproteinases (MMPs) are zinc-dependent endopeptidases belonging to the metzincin superfamily and are predominantly extracellular enzymes capable of cleaving a wide range of extracellular matrix proteins and bioactive peptides [136]. MMP-2 (EC 3.4.24.24), MMP-3 (EC 3.4.24.B6), and MMP-9 (EC. 3.4.24.35) have all been shown to possess A*β*-degrading activity *in vitro*; however, unlike ECE-1, NEP, and IDE, MMP-9 is capable of cleaving aggregated A*β* fibrils [137]. Furthermore, significant elevations in A*β* have been reported in the brains of both MMP-2 and MMP-9 knockout mice [138]. Although basal expression levels of MMPs are low in the brain, several cell types (glial, neuronal, and vascular) upregulate endogenous MMP-2, -3, and -9 expression in response to A*β* treatment [139–141]. However, the expression levels and activities of these three enzymes appeared not to differ significantly between AD and control brains and were not related to A*β* plaque load [142]. At the genetic level, the analysis of polymorphisms in the MMP-3 (-1171 5A/6A) and MMP-9 (C-156T) genes indicated that the -1171 6A MMP-3 allele (which was associated with reduced promoter activity) was associated with AD whereas the MMP-9 polymorphism was not [142].

### 4.6. Other A*β* Degrading Zinc Metalloproteinases

A*β* is clearly subject to proteolysis by a range of zinc metalloproteinases as discussed in the preceding sections of this paper. Furthermore, it should be noted that new candidate enzymes in this respect are still coming to light. For example, it has recently been reported that glutamate carboxypeptidase II (GCPII) (EC 3.4.17.21), a zinc metalloproteinase expressed in multiple tissues including the brain [143], was capable of cleaving A*β* monomers at their C-termini to produce a range of smaller fragments along with A*β*1-14 that lacked the aggregation potential and cellular toxicity of full-length A*β*. The authors also demonstrated that GCPII could degrade soluble A*β* oligomers and fibrils and could reduce plaque size in the brains of APP-presenilin1ΔE9 transgenic mice. Furthermore, in cell cultures, the overexpression of GCPII reduced the levels of secreted or exogenously supplemented A*β*-peptides. In addition to GCPII the ability of ADAMs, be it in their shed or membrane-associated forms, to degrade A*β* as opposed to their normal full-length APP substrate, remains to be established.

## 5. Concluding Remarks

**Figure 4:**
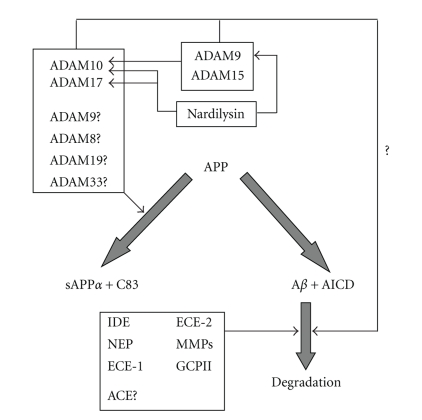
The involvement of zinc metalloproteinases in A*β* metabolism. A range of ADAMs (10, 17, 9, 8, 19, and 33) have been implicated in the nonamyloidogenic processing of APP to generate sAPP*α* and C83, although the evidence for the latter four enzymes having a direct physiological role in this respect is limited. ADAMs 9 and 15 are capable of cleaving ADAM10 and may be indirectly involved in the activation of the nonamyloidogenic pathway in this respect. Nardilysin also indirectly activates nonamyloidogenic processing of APP via an activation of ADAMs 9, 10, and 17. IDE, NEP, ECE-1, ECE-2, MMPs, GCPII and, possibly, ACE are involved directly in the degradation of A*β*, and a potential role for catalytically active ADAMs in the degradation of A*β* remains to be elucidated.

Proteolytic enzymes play a central role in AD. Whilst it is the aspartyl proteinase class that contributes directly to the formation of neurotoxic A*β*  via the amyloidogenic pathway, it is undoubtedly the zinc metalloproteinases that play the most significant roles both in the preclusion of A*β* formation via the nonamyloidogenic pathway and in the degradation of these peptides (Figure 4). Whilst much research in the AD field continues to focus on the development of inhibitors of the amyloidogenic pathway, it is clear that the upregulation of various zinc metalloproteinase activities represents a possible alternative therapeutic strategy for the treatment of the disease.
